# Genome Sequence of a Pandrug-Resistant *Pseudomonas aeruginosa* Strain, YN-1

**DOI:** 10.1128/genomeA.01280-14

**Published:** 2014-12-24

**Authors:** Hong-Li Tan, Yong Wang, Xue-Qin Cheng, Yan-Mei Huang, Wei Liu, Li-Juan Zhang

**Affiliations:** a Department of Clinical Laboratory, the Third People’s Hospital of Yunnan Province, Kunming, People’s Republic of China; b The National Institute for Communicable Disease Control and Prevention, China CDC, Beijing, People’s Republic of China; c College of Animal Science & Technology, Shihezi University, Shihezi, Xinjiang Province, People’s Republic of China

## Abstract

A highly rampant multidrug-resistant strain of *Pseudomonas aeruginosa* appeared in a hospital in Yunnan Province, China. Here, we report the genome sequence of the pandrug-resistant (PDR) *P. aeruginosa* strain recovered from a patient in 2013.

## GENOME ANNOUNCEMENT

*Pseudomonas aeruginosa* is an important opportunistic pathogen (1, 2). A multiclonal multidrug-resistant (MDR) strain is highly prevalent in a tertiary care hospital of Kunming City, the capital of Yunnan Province, China, and possesses genes associated with resistance to β-lactamases, quinolones, and aminoglycosides (3). In this study, the carbapenem-resistant pandrug-resistant (PDR) *P. aeruginosa* strain was isolated from sputum samples collected from a hospitalized patient at the Third People’s Hospital of Yunnan Province. The strain was found to be resistant to all 21 types of antibiotics tested, including penicillin, cephalosporins, aminoglycosides, quinolones, and imipenem (3). The analysis of drug resistance determinants indicated that this strain carries resistance genes, including *bla*_OXA-10_, *aac(6*’)*-Ib-cr, bla*_IMP_, *aacA4*, and *aadB*.

The genome sequence of this PDR *P. aeruginosa* strain was determined using the Illumina HiSeq 2000 sequencing platform. In total, 4,788,082 clean reads were assembled using the SOAP*denovo* assembler (http://soap.genomics.org.cn/) (4). There were 341 contigs generated for the genome, with an *N*_50_ of 56,287 bp, with the largest contig consisting of 218,487 bp. The 341 contigs were subsequently assembled into 227 scaffolds. The *N*_50_ of the scaffolds was 80,784 bp, and the length of the largest scaffold was 346,668 bp.

The length of the draft genome of the PDR *P. aeruginosa* circular chromosome was 6,980,658 bp, with a G+C content of 65.83%. The draft genome included a total of 6,657 open reading frames, accounting for 86.95% of the chromosome and with an average length of 912 bp, as predicted by Glimmer (version 3.02 [http://ccb.jhu.edu/software/glimmer/index.shtml]). The genome encodes 61 tRNAs, 34 small RNAs (sRNAs), 1 copy of 23S rRNA, and 2 copies of 16S rRNA. In addition, 9 gene islands and 5 prophages were predicted using SIGI-HMM (version 3.8) and Phast (http://phast.wishartlab.com/), respectively. Several antibiotic resistance determinants were found in this chromosome, including the carbapenem resistance genes *bla*_OXA-10_ and *bla*_IMP_, the quinolone resistance gene *aac(6*’)*-Ib-cr*, the aminoglycoside resistance genes *aacA4* and *aadB*, and class I integron genes. These genes were probably carried on the plasmid, and we will report these findings in the future.

A detailed report of our isolate will be included in a future publication, along with a full comparative analysis involving multiple published *P. aeruginosa* genomes.

### Nucleotide sequence accession number.

This whole-genome shotgun project has been deposited in the NCBI GenBank under the accession no. JRAU00000000. The version described in this paper is the first version.
